# Stoner Ferromagnetism in Hole-Doped CuM^IIIA^O_2_ with M^IIIA^ = Al, Ga, and In

**DOI:** 10.1021/acsami.1c00403

**Published:** 2021-06-21

**Authors:** Konstantina Iordanidou, Clas Persson

**Affiliations:** Centre for Materials Science and Nanotechnology, Department of Physics, University of Oslo, P.O. Box 1048, Blindern, 0316 Oslo, Norway

**Keywords:** CuAlO_2_, hole doping, defects, ferromagnetism, first-principles

## Abstract

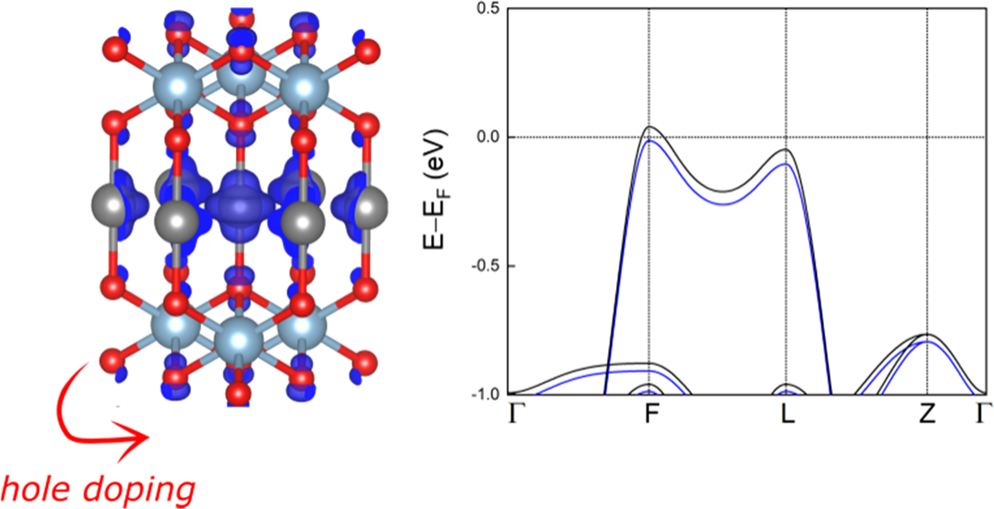

Using density functional theory calculations, we examine the effect of hole doping on the magnetic and electronic properties of CuM^IIIA^O_2_, with M^IIIA^ = Al, Ga, and In. CuM^IIIA^O_2_ nonmagnetic semiconductors switch to ferromagnetic half-metals upon hole doping. For CuAlO_2_, the nonmagnetic-to-ferromagnetic transition occurs for hole densities of ∼7 × 10^19^/cm^3^. Ferromagnetism arises from an exchange splitting of the electronic states at the valence band edge, and it can be attributed to the high-lying Cu-d states. Hole doping induced by cation vacancies and substitutional divalent dopants is also investigated. Interestingly, both vacancies and nonmagnetic divalent dopants result in the emergence of ferromagnetism.

## 1 Introduction

Ferromagnets such as iron, cobalt, and nickel, are typically metals, whereas materials with both ferromagnetic and semiconducting properties are scarce. Ferromagnetic semiconductors are attractive candidates for future spintronic devices, owing to their easy integration into the existing semiconductor devices.^1^ Over the past few decades, diluted magnetic semiconductors (DMS), i.e., nonmagnetic semiconductors doped with magnetic ions, have triggered intense interest.^2−5^ Magnetic moments mainly arise from partially filled 3d and 4f orbitals of transition metals and rare earth metals, and examples of DMS include (Ga,Mn)As, (Zn,Cr)Se, and (Pb,Eu)Te. Although Ga_1–*x*_Mn*_x_*As has been one of the most widely studied DMS, its low Curie temperature hinders its utilization in practical applications. Contrary to GaAs-based DMS, room-temperature ferromagnetism has been observed in diluted magnetic oxides such as TiO_2_, ZnO, and SnO.^6−10^ Intriguingly, high-temperature ferromagnetism in the absence of magnetic ions has been additionally reported.^11−21^ For instance, Kenmochi et al. found that carbon-doped oxides such as MgO, SrO, BaO, and CaO are ferromagnetic without any transition-metal impurities, and based on the mean-field approximation, Curie temperatures higher than room temperature have been predicted.^19−21^

Two types of diluted magnetic semiconductors with different properties have been widely studied. First, for DMS like Mn-doped GaSb with localized majority d-states deep in the valence band, ferromagnetism arises from the Zener’s p–d exchange mechanism, resulting in holes in the majority p-valence band. Such interaction is relatively weak and long-range, and the Curie temperature scales linearly with the impurity concentration. On the other hand, for DMS like Mn-doped GaN with impurity bands within the band gap, ferromagnetism arises from the Zener’s double-exchange mechanism. The magnetic couple is strong and short-ranged, and the Curie temperature scales proportionally to the square root of the impurity concentration.^22,23^

Doping of CuAlO_2_ with magnetic ions such as Mn, Fe, and Co, to form oxide-based DMS, has been intensively investigated.^24−27^ For instance, first-principles calculations revealed that (Cu,Fe)AlO_2_ and (Cu,Co)AlO_2_ are attractive candidates as high-Tc ferromagnets. Overall, CuAlO_2_ is an attractive candidate for various applications owing to its p-type conductivity (without intentional doping) and its visible light transparency.^28−30^ In general, the design of p-type transparent conductive oxides (TCO) is found to be difficult, and the difficulty arises from the nature of the VB edge, dominated by localized O-2p states.^31,32^ Holes are typically trapped at O sites and cannot migrate through the oxide, leading to poor p-type behavior. To overcome this limitation, modulation of the VB edge through hybridization of the O-2p orbitals with metal orbitals has been proposed.^31^ The discovery of p-type conductivity in CuAlO_2_ has paved the way to the discovery of other p-type TCO like CuGaO_2_ and CuInO_2_.

In this paper, using density functional theory (DFT) calculations, we examine the effect of hole doping on the magnetic properties of CuM^IIIA^O_2_ with M^IIIA^ = Al, Ga, and In, having CuAlO_2_ as a prototype material. Despite that these materials are intrinsically nonmagnetic, stable ferromagnetic phases appear for a wide range of hole densities. In addition the possibility of inducing hole doping and also ferromagnetism by cation vacancies and substitutional divalent dopants is investigated. Contrary to previous studies, we focus on doping with nonmagnetic ions. Magnetic ions typically lead to localized magnetic moments instead of ferromagnetic phases, and materials with localized magnetic moments are likely to present low spin polarization of the charge carriers, hindering their utilization in future spintronic devices. In our work, we find that nonmagnetic divalent dopants result in the emergence of ferromagnetism, and ferromagnetism can be attributed to the high-lying Cu-d states, dominating over the O-p states at the VB edge.

## 2 Methods and Computational Details

We perform spin-polarized DFT calculations using the Vienna Ab initio Simulation Package (VASP).^33,34^ For the exchange correlation functional, the generalized gradient approximation introduced by Perdew, Burke, and Ernzerhof (PBE) is used.^35^ The electron–ion interaction is described by the projector augmented wave (PAW) method.^36^ For the unit cells, both the atomic positions and the volume are optimized, through the conjugate gradient method, with 10^–8^ eV energy convergence criteria and 10^–2^ eV/Å force convergence criteria. The kinetic energy cutoff is set to 600 eV, and the Brillouin zone is sampled by a 16 × 16 × 16 *k*-point grid for the atomic relaxations, whereas a 24 × 24 × 24 *k*-point grid is used for the density of states (DOS) calculations.

To achieve hole doping, we change the number of electrons of the unit cell and we add a compensating opposite charge background. Since the magnetic instabilities sensitively depend on the DOS near the Fermi level, a very dense grid of 48 × 48 × 48 *k*-points was used for the calculations of the magnetic moments. Hybrid functional calculations were also performed, using the Heyd–Scuseria–Ernzerhof (HSE) functional and the standard range-separation and mixing (0.25 Fock exchange) parameters.^37^ Due to computational issues, for the hybrid functional calculations, an 8 × 8 × 8 *k*-point grid was adopted.

Finally, for the defective systems, we used supercells consisting of 192 atoms in total. The Brillouin zone was sampled by a 2 × 2 × 2 *k*-point grid for the atomic relaxations, whereas a 4 × 4 × 4 *k*-point grid was used for the DOS calculations.

## 3 Results

### 3.1 Ferromagnetism in Hole-Doped CuM^IIIA^O_2_

**Figure 1:**
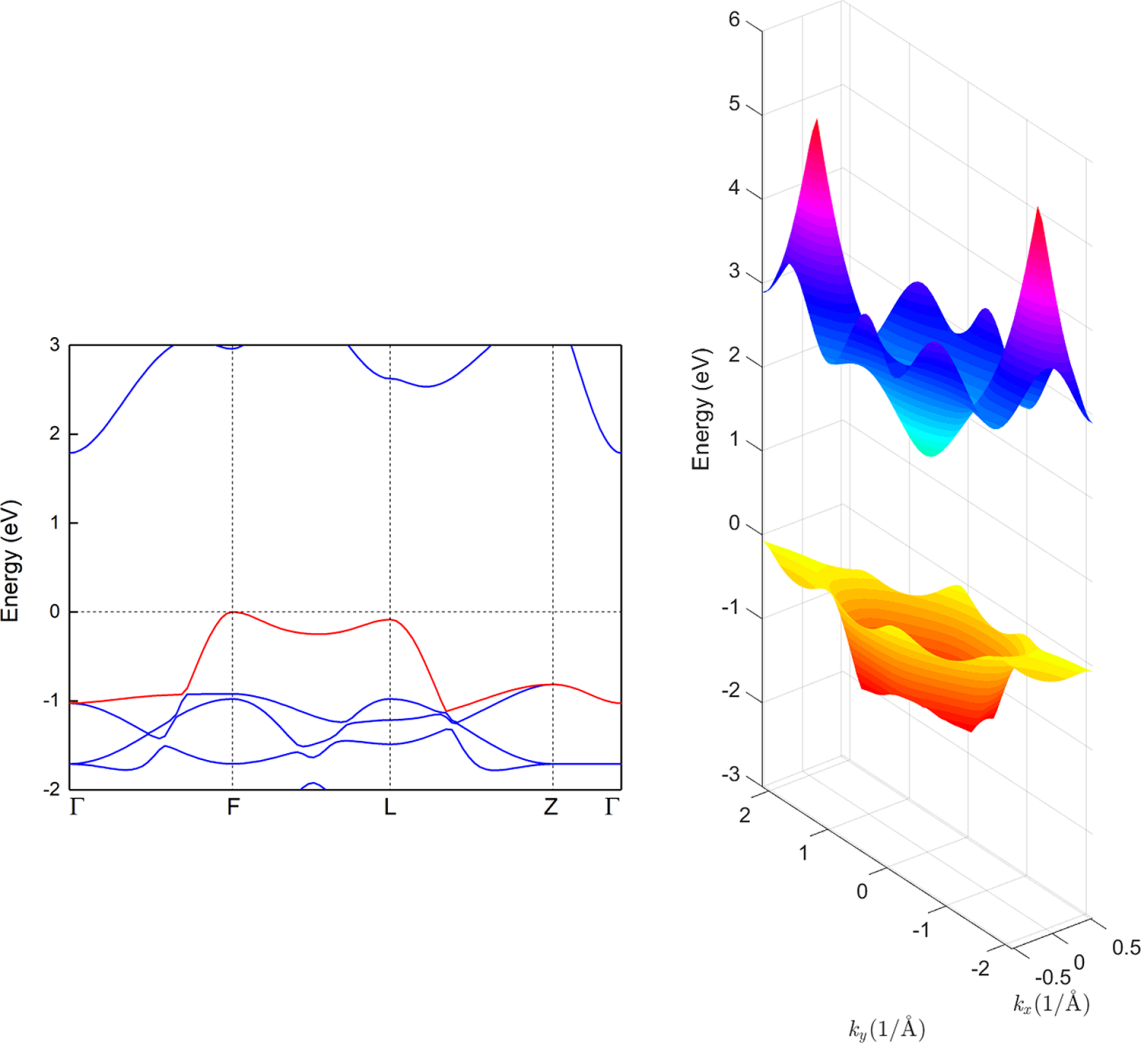
Two-dimensional band structure of CuAlO_2_ unit cell, and three-dimensional band structure for the topmost valence band and the bottommost conduction band. The three-dimensional band structure is obtained using the VASPKIT code.^56^ The energies refer to the valence band maximum. The high-symmetry *k*-points are Γ (0, 0, 0), *F* (0.5, 0.5, 0), *L* (0.5, 0, 0), and *Z* (0.5, 0.5, 0.5). The topmost VB is highlighted in red.

**Figure 2:**
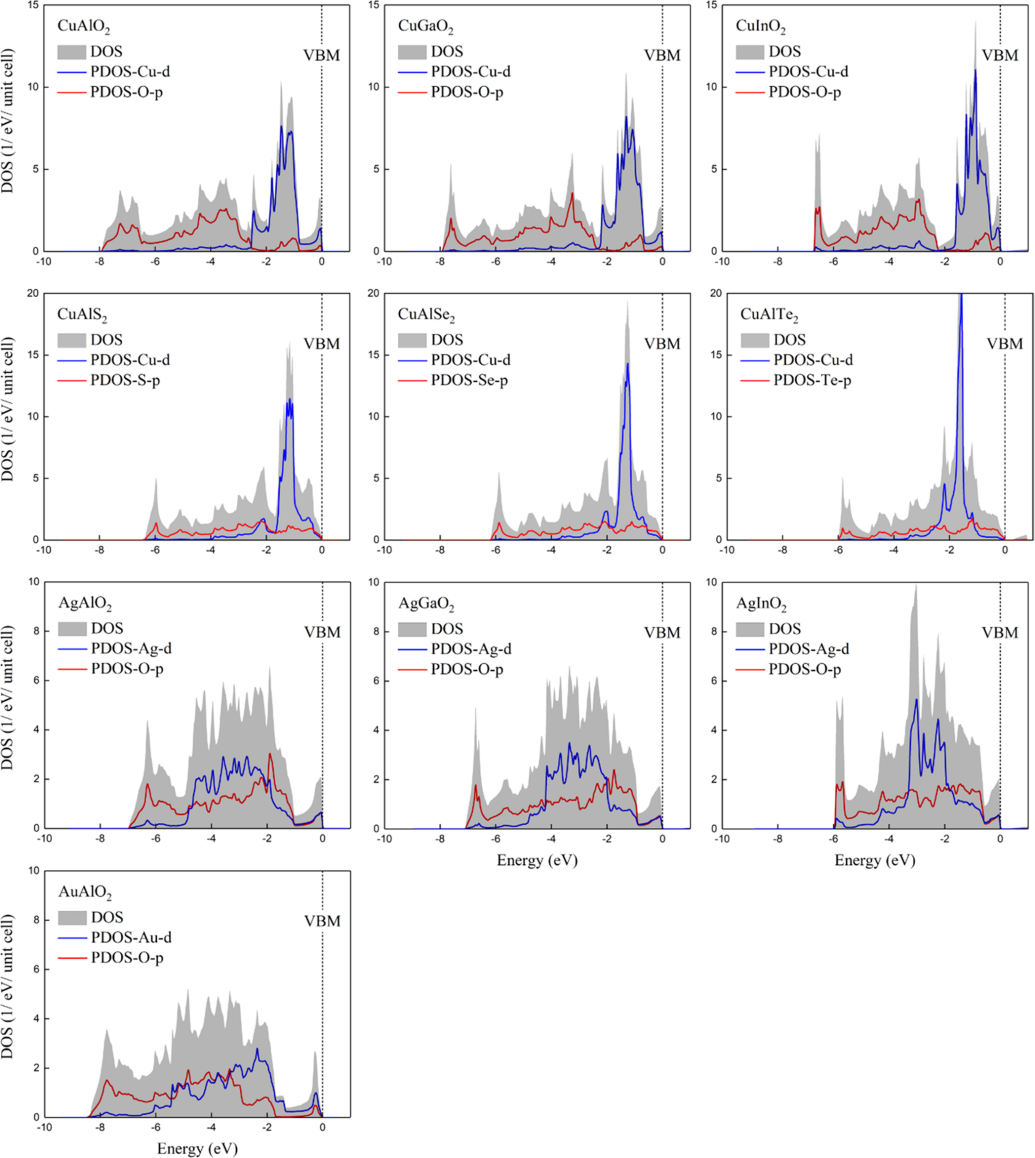
Total and projected valence band DOS of CuM^IIIA^O_2_ with M^IIIA^ = Al, Ga, and In (first-row panels), CuAlY_2_ with Y = S, Se, and Te (second-row panels), AgM^IIIA^O_2_ (third-row panels), and AuAlO_2_ (fourth-row panel). A Lorentzian broadening of 0.02 eV is used. The energies refer to the valence band maximum. The nonparticipating M^IIIA^ states are not displayed.

CuAlO_2_ crystallizes in the delafossite structure (space group: *R*3̅*m*), where each Cu atom is bonded to two O atoms forming O–Cu–O dumbbells, whereas each Al atom is bonded to six O atoms forming distorted octahedra. The Cu–O and Al–O bond lengths are found to be 1.885 and 1.925 Å, respectively, whereas for the optimized nonprimitive unit cell, the in-plane and out-of-plane lattice constants are *a* = *b* = 2.880 and *c* = 17.119 Å, in excellent agreement with experimental observations.^38^ A summary of the structural parameters of CuM^IIIA^O_2_ with M^IIIA^ = Al, Ga, and In, in the delafossite structures is given in Table S1 in the Supporting Information. As shown in Figure 1, CuAlO_2_ is an indirect band gap semiconductor with *E*_g_ = 1.79 eV, in agreement with previously reported DFT-PBE calculations.^39^ Using HSE calculations, a gap opening of about 1.8 eV is found. The valence band (VB) top and the conduction band (CB) bottom are located at the *F* and Γ points, respectively, and the VB top is mainly derived from Cu-d orbitals along with a smaller contribution of O-p orbitals, as observed in Figure 2. Notably, the dispersion of the topmost VB looks like a slightly asymmetric inverted Mexican hat, where the band extrema are located at the *F* and *L* points, and the energy difference between the *F* and *L* points is 85 meV. This peculiar VB dispersion leads to a sharp high peak of the DOS near the VB edge. By shifting the Fermi level close to this peak, the Stoner criterion is fulfilled and the system becomes ferromagnetic.^40−47^ Hence, hole-doped CuAlO_2_ could be a ferromagnetic material.

**Figure 3:**
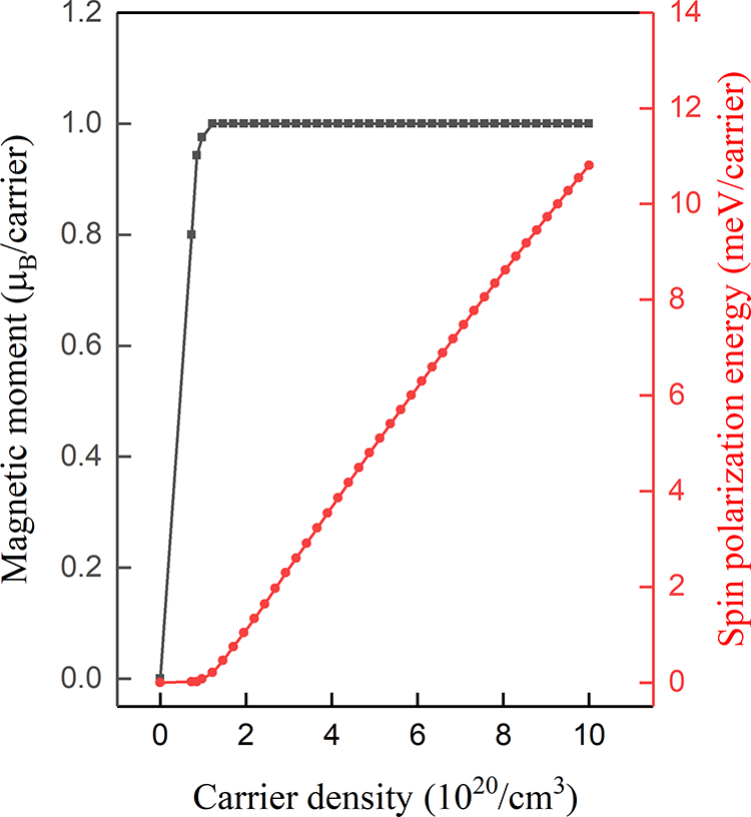
Carrier-dependent magnetic moment and spin polarization energy for CuAlO_2_.

To verify this assumption, we consider charged systems. Upon hole doping, a very slight shortening of the Cu–O bond lengths is observed, whereas the Al–O bond lengths present the opposite trend (see Supporting Information Figure S1). The magnetic moments and the spin polarization energies as a function of the injected holes are computed. The spin polarization energies are obtained through the equation *E*_SP_ = *E*_NM_ – *E*_FM_, where *E*_NM_ and *E*_FM_ are the energies of the nonmagnetic and ferromagnetic phases, respectively. Using this definition, positive spin polarization energies indicate stable ferromagnetic phases. As shown in Figure 3, a ferromagnetic state spontaneously emerges for hole densities of ∼7 × 10^19^/cm^3^. The magnetic moment per carrier rapidly reaches the saturation value of 1 μ_B_, and it remains unchanged for hole densities up to ∼1 × 10^22^/cm^3^. Further increase of the hole density leads to the reduction of the magnetic moment. On the other hand, the spin polarization energy per carrier strongly depends on the doping level. At the relatively low doping level, it increases by increasing the density. For *p* = 6 × 10^21^/cm^3^, it reaches the maximum value of ∼46 meV/carrier, which is larger compared to the corresponding values in similar investigations,^44,45^ whereas for larger hole densities, it gradually decreases until it becomes zero. Contrary to the magnetic moment per carrier, no plateau region is observed in the spin polarization energy per carrier.

Owing to the self-interaction error, semilocal functionals tend to overdelocalize electrons, and for systems with d electrons, this can be particularly problematic. Hartree–Fock and semilocal functionals typically present an opposite self-interaction error. Thus, hybrid functionals, which use a fraction of the Hartree–Fock and a fraction of the semilocal functional, tend to reduce the self-interaction error.^48^ In our work, hybrid functional calculations have been additionally performed for the hole-doped systems. Notably, using HSE calculations, the peculiar Mexican-hat-like valence band dispersion is retained and the energy difference between the *F* and *L* points is 91 meV, i.e., slightly larger compared to the PBE calculations. The magnetic properties of CuAlO_2_ at different hole densities in the range of ∼10^19^–10^22^/cm^3^ are examined, and similar to our PBE calculations, hole-doped CuAlO_2_ is found to be ferromagnetic. It is worth noting that the so-called pseudopotential self-interaction method (pseudo-SIC) has been widely used to eliminate self-interaction errors in standard DFT calculations.^49−51^ For instance, the self-interaction-corrected electronic structures of ZnO-based DMS have been reported. Compared to those computed using standard DFT, a larger band gap and a qualitatively different description of the transition-metal d states have been observed.

**Figure 4:**
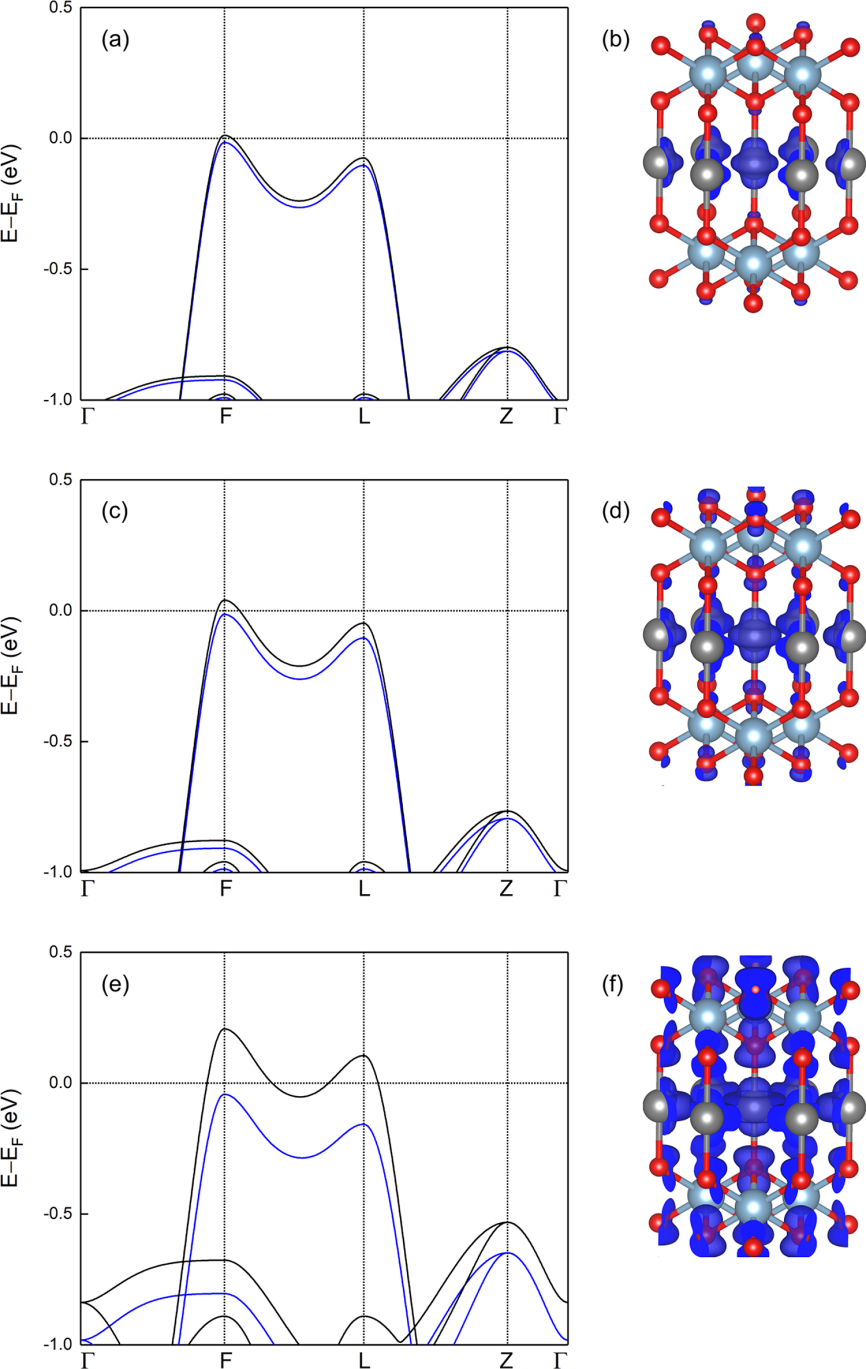
Electronic band structures (left) and spin density plots (right) of CuAlO_2_ unit cells at different hole densities: (a, b) 5.0 × 10^20^/cm^3^, (c, d) 1.0 × 10^21^/cm^3^, and (e, f) 5.0 × 10^21^/cm^3^. The blue and black lines correspond to spin-up-like and spin-down-like states. The high-symmetry *k*-points are Γ (0, 0, 0), *F* (0.5, 0.5, 0), *L* (0.5, 0, 0), and *Z* (0.5, 0.5, 0.5). The gray, light blue, and red spheres correspond to Cu, Al, and O atoms. The isosurface is set to 0.0005 electrons per Bohr^3^.

As a next step, we compute the electronic band structures and the spin density plots of the charged systems. The spin density plots are obtained by the spin-up and spin-down charge density differences. As shown in Figure 4, an exchange splitting of the electronic states near the Fermi level is observed at *p* = 5.0 × 10^20^/cm^3^. The energy difference between the spin-up and spin-down valence band maxima significantly increases, by increasing the hole density. On the other hand, we observe a shift of the Fermi level toward the lower energies. This results in a half-metallic behavior, i.e., the spin-up and spin-down states present semiconducting and metallic nature, respectively, allowing a fully polarized spin transport. The magnetization mainly arises from Cu-d orbitals along with O-p orbitals. For instance, for *p* = 1 × 10^21^/cm^3^, the magnetization of Cu, Al, and O atoms is 86, 0, and 14% of the total magnetization, and very similar results are found for other hole densities. These results are consistent with the observation that the magnetic behavior is originated from the VB edge, which mainly consists of Cu-d states with a smaller contribution of O-p states.

Finally, we perform calculations to estimate the Curie temperature of CuAlO_2_ at various hole densities. Our calculations are based on the mean-field evaluation of the magnetic moment by minimizing the electronic free energy of the system at specific temperatures.^52^ To introduce the finite temperature, we modify the smearing factor (σ = *k*_B_*T*) of the Fermi–Dirac distribution. At the relatively low doping level, the Curie temperature is below room temperature. By increasing the hole density, the Curie temperature increases, and at the high doping level, it becomes much larger than room temperature (see Supporting Information Figure S2). It should be noted that the Curie temperatures can be significantly overestimated using this approach, and the real values can be lower compared to the predicted values by as much as a factor of 2.^53^

Overall, our findings revealed that CuAlO_2_ could be used for future spintronic devices like the spin field effect transistors (spin FETs) and the magnetic tunnel junctions (MTJs). We found that the injection of holes in CuAlO_2_ could control/manipulate its electronic and magnetic properties. Control of magnetism through an applied voltage offers various advantages like ultralow power dissipation and reversibility, and electrically controlled ferromagnetism is highly promising for achieving optimal spintronic devices.

Besides CuAlO_2_, other group-IIIA Cu-based oxides are examined, namely, CuGaO_2_ and CuInO_2_. Both CuGaO_2_ and CuInO_2_ present an asymmetric Mexican-hat-like VB dispersion, and the energy difference between the band extrema located at the *F* and *L* points are 149 and 83 meV, respectively (see Supporting Information Figures S3 and S4). Upon hole doping, a slight shortening of the Cu–O bond lengths along with a slight elongation of the Ga–O and In–O bond lengths is observed (see Supporting Information Figure S5). A nonmagnetic-to-ferromagnetic transition occurs at *p* = 1 × 10^20^/cm^3^ and *p* = 3 × 10^19^/cm^3^ for Ga- and In-based compounds, respectively, and half-metallicity has been additionally observed (see Supporting Information Figures S6–S8). Both CuGaO_2_ and CuInO_2_ return to the nonmagnetic state for a hole density of ∼4 × 10^22^/cm^3^.

Furthermore, we examine if the corresponding chalcogenides CuAlY_2_, where Y = S, Se, and Te, in the delafossite structures, are also promising for realizing a ferromagnetic phase upon hole doping. First, we introduce a measure for the hybridization strength between the projected orbital l of atom α and the projected orbital l′ of atom α′, using the normalized cross-correlation of DOS *g*(*E*)
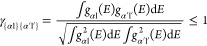
1In the entire VB region, the hybridization strengths between Cu-d and Y-p orbitals are found to be 0.50, 0.50, and 0.41 for CuAlS_2_, CuAlSe_2_, and CuAlTe_2_, respectively, whereas the corresponding value for CuAlO_2_ is only 0.21. In addition, contrary to the oxides where the VB edge is dominated by Cu-d states, for the sulfides and selenides, a strong hybridization of Cu-d and Y-p states is observed, whereas for tellurides, the VB edge is dominated by Te-p states, as shown in Figure 2. Thus, going from oxygen to tellurium compounds, the Cu-d character of the VB edge is found to decrease. The different nature of the VB edge in chalcogenides compared to the oxides leads to the absence of the peculiar Mexican-hat-like VB dispersion and the subsequent absence of the sharp high peak of the DOS at the VB edge. Thus, CuAlY_2_, with Y = S, Se, and Te is expected to be nonmagnetic upon hole doping. To verify this assumption, additional calculations were performed and we found that hole doping in the range of 1 × 10^19^–1 × 10^22^/cm^3^ results in no magnetization in CuAlY_2_.

AgM^IIIA^O_2_, where M^IIIA^ = Al, Ga, and In, as well as AuAlO_2_ are also discussed. The hybridization strengths between Ag-d and O-p orbitals, in the entire VB region, are 0.83, 0.79, and 0.78 for M^IIIA^ = Al, Ga, and In, respectively, whereas the corresponding values for their Cu-based counterparts are only 0.21, 0.22, and 0.20. In addition, going from Cu- to Ag-based compounds, the metal-d character of the VB edge significantly decreases, leading to a less sharp peak of the DOS at the VB edge, as shown in Figure 2. Accordingly, for Ag-based compounds, the asymmetry in the Mexican-hat-like VB dispersion significantly increases and the energy differences between the band extremum located at the *F* and *L* points are 258, 428, and 340 meV for AgAlO_2_, AgGaO_2_, and AgInO_2_, respectively. This should lead to a much less promising behavior of hole-doped AgM^IIIA^O_2_ compounds compared to CuM^IIIA^O_2_. Indeed, our calculations reveal that for AgM^IIIA^O_2_, no ferromagnetism is observed for hole densities up to ∼1 × 10^22^/cm^3^.

Interestingly, AuAlO_2_ presents an intermediate behavior between CuAlO_2_ and AgAlO_2_. The hybridization strength between Au-d and O-p orbitals in the entire VB region is 0.76, whereas the VB edge has a weaker (stronger) d-character compared to CuAlO_2_ (AgAlO_2_), as shown in Figure 2. Furthermore, for AuAlO_2_, the asymmetry in the Mexican-hat VB is larger (smaller) compared to CuAlO_2_ (AgAlO_2_) and the energy difference between the *F* and *L* points is 204 meV. We find that AuAlO_2_ becomes ferromagnetic for only a narrow range of hole densities, i.e., ∼5 × 10^21^ < *p* < 9 × 10^21^/cm^3^, and for the optimal hole density, the magnetic moment is less than 1 μ_B_/carrier. A summary of the key electronic- and magnetic-related characteristics for all studied systems is shown in Table 1. A measure of the d-state strength in the VB edge is introduced using the formula
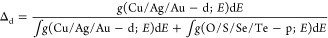
2and for this measure, we use an integration region of 0.3 eV below the VB top.

**Table 1:** Electronic- and Magnetic-Related Properties for All Studied Systems^a^

system	Δ*E*_FL_ (meV)	γ	Δ_d_	*p*_NM-FM_ (cm^–3^)
CuAlO_2_	85	0.21	0.81	7 × 10^19^
CuGaO_2_	149	0.22	0.84	1 × 10^20^
CuInO_2_	83	0.20	0.85	3 × 10^19^
AgAlO_2_	258	0.83	0.50	
AgGaO_2_	428	0.79	0.49	
AgInO_2_	340	0.78	0.52	
AuAlO_2_	204	0.76	0.68	5 × 10^21^
CuAlS_2_		0.50	0.42	
CuAlSe_2_		0.50	0.32	
CuAlTe_2_		0.41	0.21	

a Δ*E*_FL_ refers to the energy difference between the *F* and *L* high-symmetry *k*-points, γ is the hybridization strength between the metal-d and oxygen/chalcogen-p states in the VB, Δ_d_ is the d-state strength in the VB edge, and *p*_NM-FM_ corresponds to the hole density, where a nonmagnetic-to-ferromagnetic transition occurs. For the estimation of *p*_NM-FM_, calculations for hole densities up to ∼1 × 10^22^/cm^3^ are performed.

### 3.2 Hole Doping Induced by Intrinsic Defects in CuAlO_2_

As a next step, we examine the possibility of inducing hole doping and also ferromagnetism by intrinsic defects. In our study, we focus on cation vacancies (V_Cu_ and V_Al_) in the neutral charge state. Regarding the structural properties, by removing a Cu atom, two undercoordinated O atoms are found. These atoms exhibit a slight outward relaxation by 2.1%, leading to shorter Al–O bond lengths of 1.852 Å. Accordingly, Cu atoms surrounding the missing lattice site display an outward relaxation of ∼1.3%. Contrary to Cu vacancy, which causes only a minor distortion of the lattice, Al vacancy induces a larger deformation. In particular, threefold-coordinated O atoms, neighboring the vacant site, relax outward by 10.5%, resulting in shorter Cu–O bond lengths of 1.805 Å.

We compute the defect formation energies through the equation *E*_for_ = *E*_tot_ (CuAlO_2_:V_A_) – *E*_tot_ (CuAlO_2_) + μ_A_, where *E*_tot_ (CuAlO_2_:V_A_) and *E*_tot_ (CuAlO_2_) are the energies of the defective and defect-free structures, respectively, whereas μ_A_ is the chemical potential of the removed cation atom, which refers to the corresponding solid structure. Cu vacancy formation energy is found to be 0.9 eV, whereas the formation energy of Al vacancy is as high as 8.4 eV, in agreement with previously reported theoretical calculations.^54^

**Figure 5:**
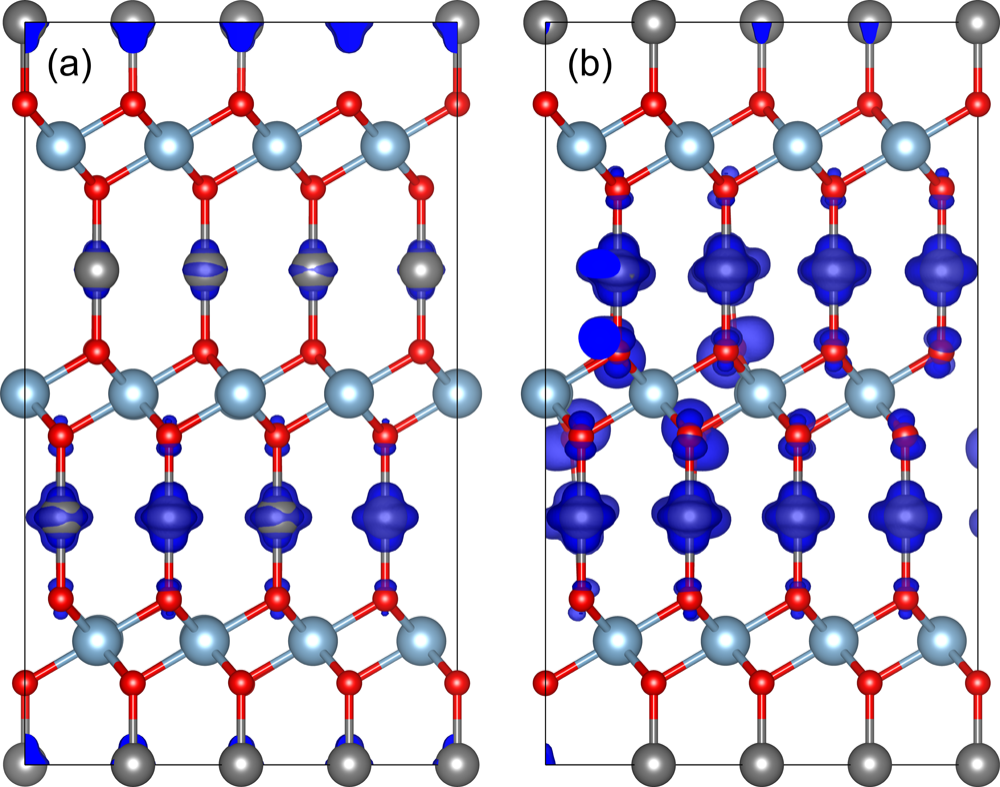
Spin density plots of (a) Cu-deficient and (b) Al-deficient CuAlO_2_. The isosurface is 0.001 electrons per Bohr^3^.

When a Cu atom is removed, one hole is introduced in the system, whereas the removal of an Al atom corresponds to three induced holes in the system. The corresponding hole densities are about 5 × 10^20^ and 15 × 10^20^/cm^3^ for Cu- and Al-deficient systems, respectively. Cu vacancy leads to a total magnetization of 1 μ_B_, whereas Al vacancy results in a total magnetic moment of 3 μ_B_, which is equivalent to the number of holes. For the case of Cu vacancy, the local magnetic moments of the nearest-neighboring Cu sites are ∼0.05 μ_B_, whereas all other Cu sites present nonzero magnetic moments up to 0.04 μ_B_. In addition, the total Cu, Al, and O magnetizations are found to be ∼0.9, 0.0, and 0.1 μ_B_, respectively. At the same hole concentration, for the hole-injected CuAlO_2_, the Cu, Al, and O magnetizations are also 90, 0, and 10% of the total magnetization, respectively. Next, the relaxation effect on the magnetic properties is investigated. In agreement with the previous results, the unrelaxed defective structure has a total magnetic moment of 1 μ_B_ and the distribution of the local magnetic moments is very similar. Figure 5 shows the spin density plots for both vacancy defects. The spin polarization energies of Cu- and Al-deficient systems are found to be ∼7 and 93 meV, respectively, and the positive values reveal that the ferromagnetic phases are favorable compared to the nonmagnetic phases. Test calculations for singly negatively charged Cu vacancies and triply negatively charged Al vacancies are also performed. The charged defective systems are found to be nonmagnetic, confirming that the induced holes are responsible for the emergence of ferromagnetism.

**Figure 6:**
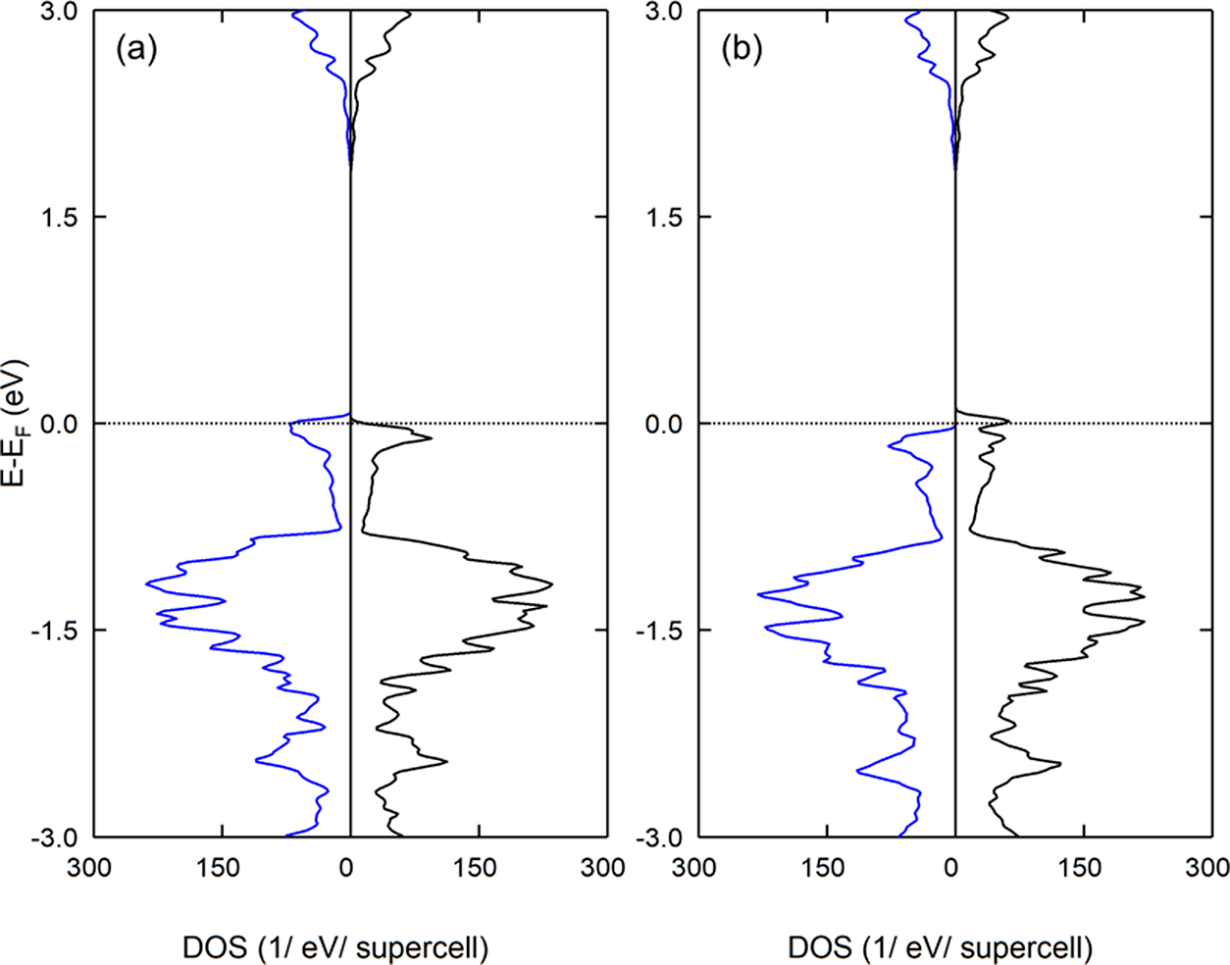
DOS of (a) Cu-deficient and (b) Al-deficient CuAlO_2_. A Lorentzian broadening of 0.02 eV is used. The blue and black lines correspond to spin-up-like and spin-down-like DOS.

Regarding the electronic properties, Figure 6 shows the DOS of the defective CuAlO_2_. For both cation vacancies, no deep gap states are observed. Due to the induced holes, the Fermi level is shifted within the VB, leading to an asymmetry between the spin-up and spin-down states, and both systems are found to be half-metals. Obviously, the Fermi-level shift for the Al-deficient system is larger compared to the Cu-deficient one.

### 3.3 Hole Doping Induced by Extrinsic Defects in CuAlO_2_

Next, we study the substitution of Al atoms by group-IIA and group-IIB atoms, namely, X = Mg, Ca, Zn, and Cd. Note that Al atoms have three valence electrons, whereas group-IIA and group-IIB have only two valence electrons. Thus, these substitutional dopants are attractive alternatives for inducing hole doping and also ferromagnetism in CuAlO_2_. Concerning the structural properties, oxygens surrounding the Mg, Ca, Zn, and Cd dopants display an outward relaxation of ∼6.1, 14.1, 8.0, and 14.8%, resulting in elongated Mg–O, Ca–O, Zn–O, and Cd–O bond lengths of 2.04, 2.20, 2.08, and 2.21 Å, respectively. We compute the dopant formation energies through the equation *E*_for_ = *E*_tot_ (CuAlO_2_:X) – *E*_tot_ (CuAlO_2_) + μ_Al_ – μ_X_, where *E*_tot_ (CuAlO_2_:X) and *E*_tot_ (CuAlO_2_) are the energies of the doped and undoped structures, whereas μ_Al_ and μ_X_ are the chemical potentials of Al and dopant atoms, respectively, which refer to the corresponding solid structures. The formation energies are found to be 1.7, 2.8, 4.8, and 6.7 eV for Mg-, Ca-, Zn-, and Cd-doped systems, respectively.

**Figure 7:**
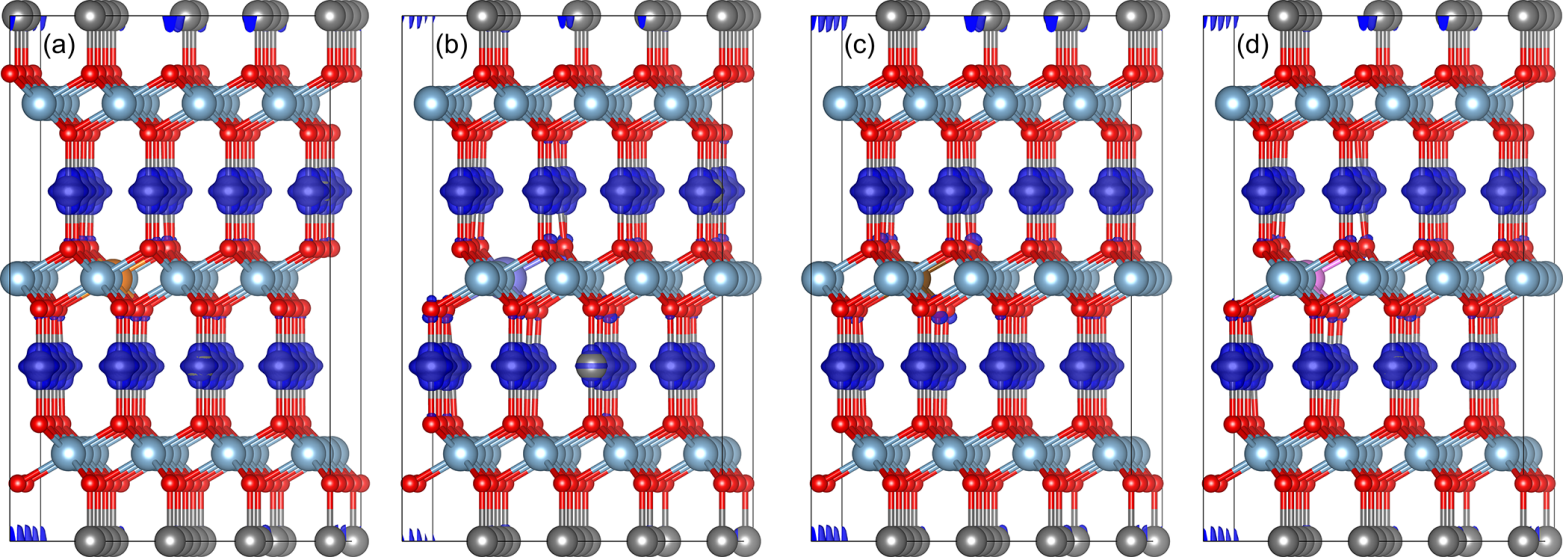
Spin density plots of doped CuAlO_2_ (a) Mg-on-Al site, (b) Ca-on-Al site, (c) Zn-on-Al site, and (d) Cd-on-Al site. The orange, purple, brown, and pink spheres correspond to Mg, Ca, Zn, and Cd atoms. The isosurface is set to 0.001 electrons per Bohr^3^.

**Figure 8:**
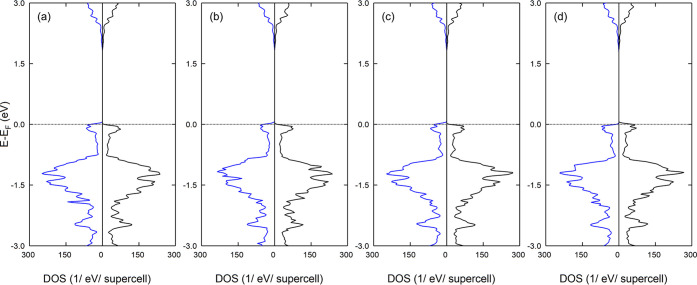
DOS of doped CuAlO_2_ (a) Mg-on-Al site, (b) Ca-on-Al site, (c) Zn-on-Al site, and (d) Cd-on-Al site. A Lorentzian broadening of 0.02 eV is used. The blue and black lines correspond to spin-up-like and spin-down-like DOS.

Taking Mg-doped CuAlO_2_ as an example, the total magnetic moment is found to be 1 μ_B_, i.e., equivalent to the number of holes. The 32 Cu atoms, which sandwich the dopant-containing Al plane, present local magnetic moments of about 0.02–0.04μ_B_, whereas the remaining 16 Cu atoms present negligible magnetic moments. Magnetization is found to be less localized in plane, compared to the out-of-plane direction. The total Cu and O magnetizations are found to be ∼ 0.9 and 0.1 μ_B_, respectively, whereas the dopant atom and all Al atoms have zero magnetic moments. Remarkably, our findings are different from those reported in similar investigations, where the magnetic moments are found to be strongly localized in the impurity atoms.^44^ Our theoretical calculations are also in good agreement with recent experimental observations, where CuAl_1–*x*_Mg*_x_*O_2_ with *x* = 0.05 is found to be ferromagnetic at room temperature.^55^ Similar results are also obtained for the magnetic moments for the other group-IIA- and group-IIB-doped systems, and their spin density plots are shown in Figure 7. For all doped systems, positive spin polarization energies are found and their values are 7.2, 6.3, 7.3, and 7.2 meV for Mg, Ca, Zn, and Cd dopants, respectively. Concerning the electronic properties, no deep gap states are observed, and an obvious shift of the Fermi level within the VB along with half-metallicity is observed, as shown in Figure 8.

## 4 Conclusions

Using density functional theory calculations, we examined the electronic and magnetic properties of CuM^IIIA^O_2_ with M^IIIA^ = Al, Ga, and In, upon hole doping. Our simulations revealed a nonmagnetic-to-ferromagnetic transition for the hole-doped systems. Ferromagnetism arises from an exchange splitting of the electronic states at the valence band edge, and it can be attributed to the high-lying Cu-d states dominating over the O-p states. Besides ferromagnetism, half-metallicity has been additionally observed, i.e., the spin-up and spin-down states present semiconducting and metallic behavior, respectively, allowing a fully polarized spin transport. The possibility of inducing hole doping and a subsequent ferromagnetic order by cation vacancies was also investigated. We found that Cu vacancy presents a formation energy lower than 1 eV and leads to a total magnetization of 1 μ_B_. The local magnetic moments of the nearest-neighboring Cu sites are ∼0.05 μ_B_, whereas all other Cu sites present nonzero magnetic moments up to 0.04 μ_B_. Besides cation vacancies, group-IIA and group-IIB atoms replacing Al atoms were examined. Interestingly, these nonmagnetic divalent dopants also result in the emergence of ferromagnetism.

## Supporting Information Available

Bond length variations for hole-doped CuM^IIIA^O_2_; temperature-dependent magnetic moment for CuAlO_2_; band structures of CuGaO_2_ and CuInO_2_; carrier dependent magnetic moment and spin polarization energy for CuGaO_2_ and CuInO_2_; band structures of hole-doped CuGaO_2_ and CuInO_2_; and structural properties of CuM^IIIA^O_2_ (PDF)
