# Supplementary material for: Stoner Ferromagnetism in Hole-Doped CuMIIIAO2 with MIIIA = Al, Ga, and In
Source: ACS Appl Mater Interfaces. 2021 Jun 21;13(25):29770–9. doi: 10.1021/acsami.1c00403 (PMC8289245; doi:10.1021/acsami.1c00403)
Supplement: Supplementary file 1 — am1c00403_si_001.pdf [file am1c00403_si_001.pdf]

Supporting Information

Stoner ferromagnetism in hole-doped $CuM^{IIIA}O_2$ with M^{IIIA}= Al, Ga, and In

Konstantina Iordanidou* and Clas Persson

Centre for Materials Science and Nanotechnology, Department of Physics, University of Oslo, P.O. Box 1048 Blindern, NO-0316 Oslo, Norway

*Email: konstantina.iordanidou@smn.uio.no

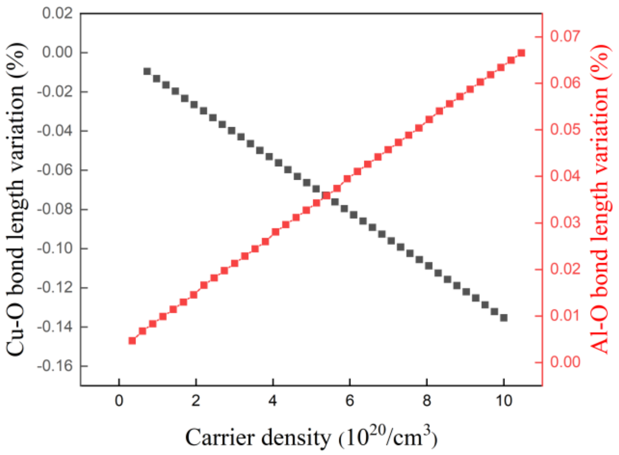

Figure S1: Cu-O and Al-O bond length variations as a function of the carrier density for $CuAlO_2$.

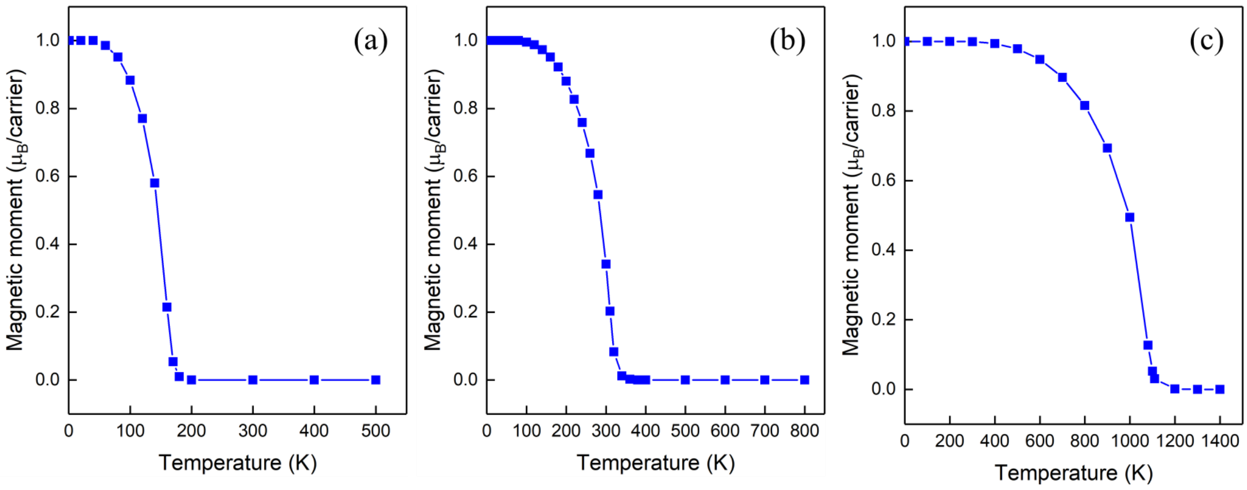

Figure S2: Temperature-dependent magnetic moment for $CuAlO_2$ at different hole densities (a) $5.0 \times 10^{20}/\mathrm{cm}^3$, (b) $1.0 \times 10^{21}/\mathrm{cm}^3$, and (c) $5.0 \times 10^{21}/\mathrm{cm}^3$.

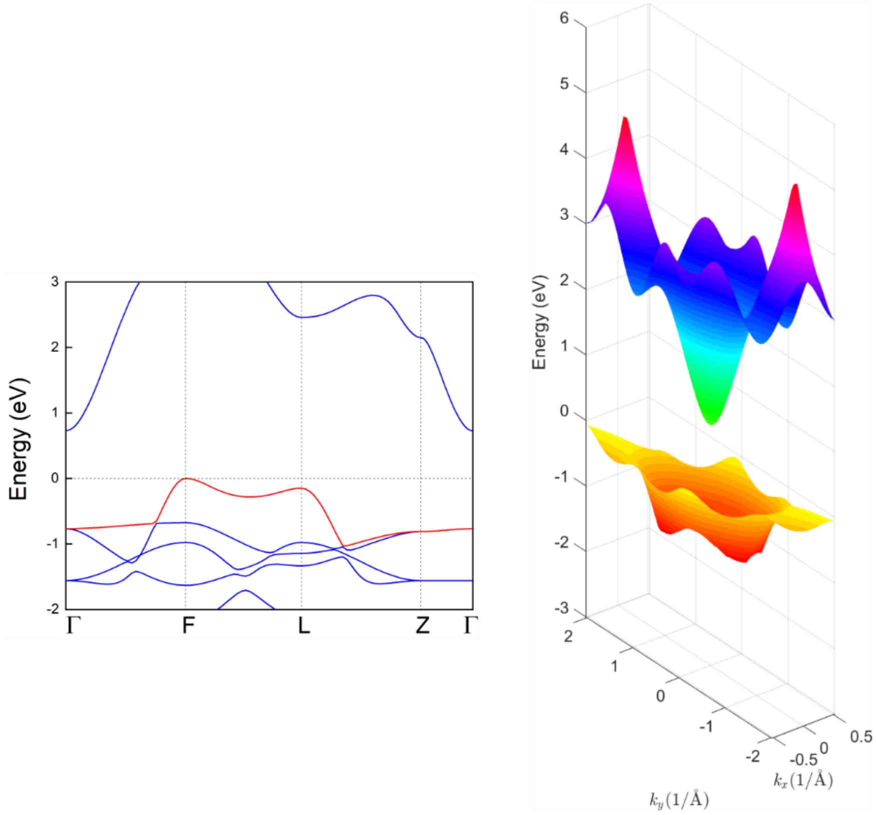

Figure S3: Two-dimensional band structure of $CuGaO_2$ unit cell, and three-dimensional band structure for the topmost valence band and the bottommost conduction band. The energies refer to the VB maximum, and the high-symmetry k-points are Γ (0, 0, 0), F (0.5, 0.5, 0), L (0.5, 0, 0), Z (0.5, 0.5, 0.5). The topmost VB is highlighted in red.

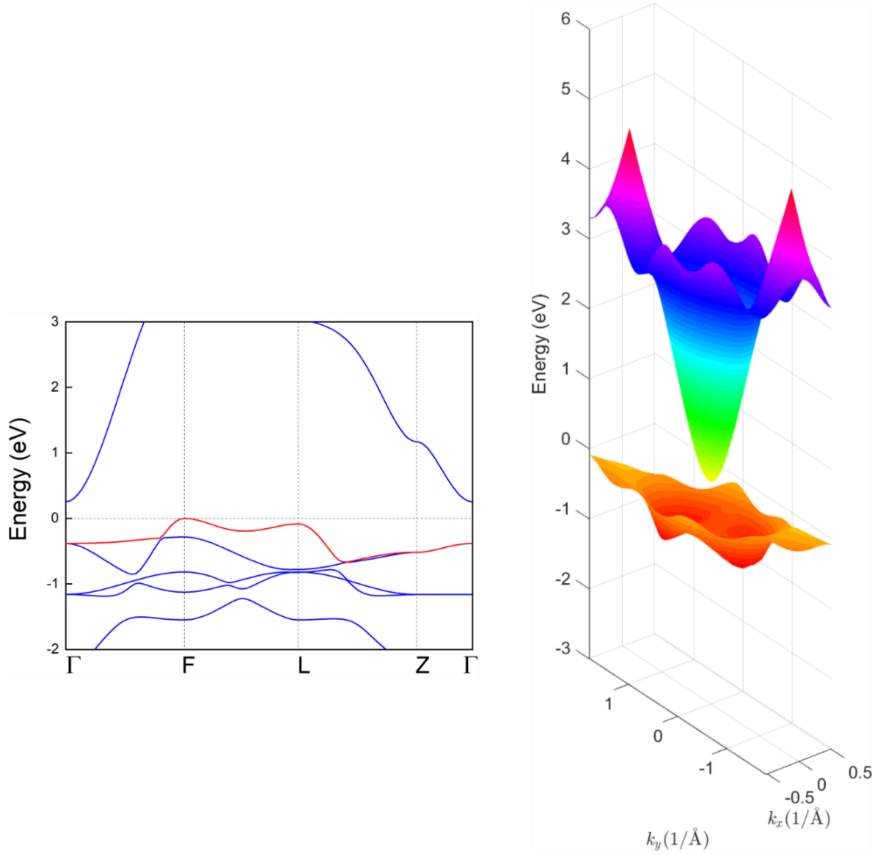

Figure S4: Two-dimensional band structure of $CuInO_2$ unit cell, and three-dimensional band structure for the topmost valence band and the bottommost conduction band. The energies refer to the VB maximum, and the high-symmetry k-points are Γ (0, 0, 0), F (0.5, 0.5, 0), L (0.5, 0, 0), Z (0.5, 0.5, 0.5). The topmost VB is highlighted in red.

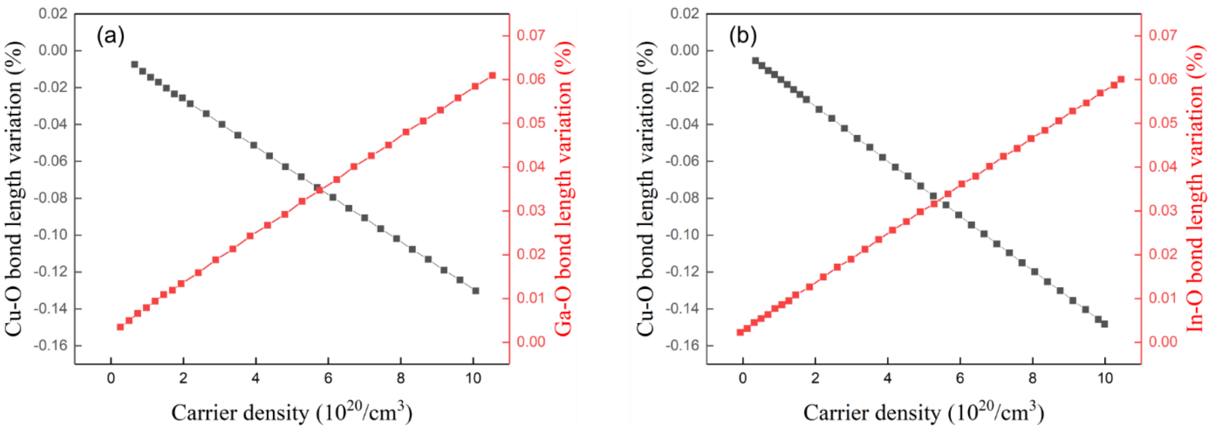

Figure S5: Cu-O and M^{IIIA}-O bond length variations as a function of the carrier density for (a) $CuGaO_2$, and (b) $CuInO_2$.

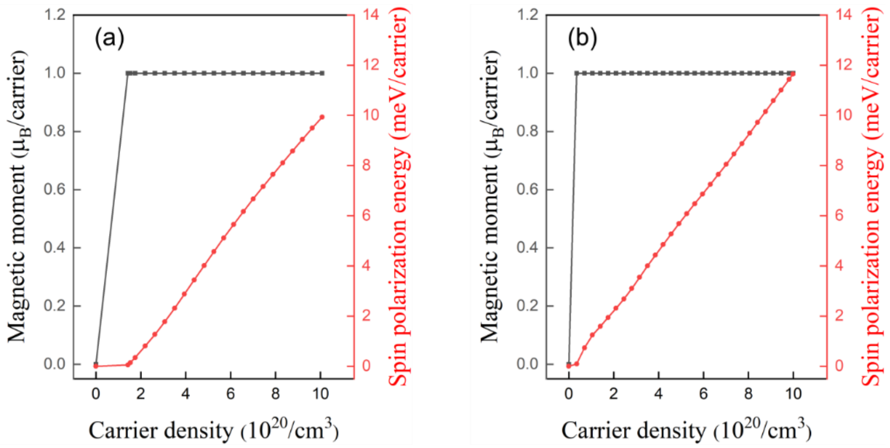

Figure S6: Carrier-dependent magnetic moment and spin polarization energy for (a) $CuGaO_2$, and (b) $CuInO_2$.

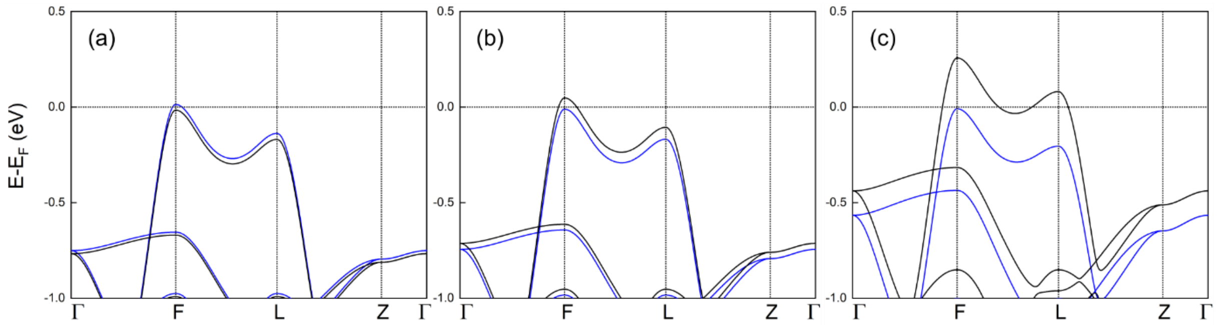

Figure S7: Electronic band structures of $CuGaO_2$ unit cells at different hole densities (a) $5.0 \times 10^{20}/\mathrm{cm}^3$, (b) $1.0 \times 10^{21}/\mathrm{cm}^3$, and (c) $5.0 \times 10^{21}/\mathrm{cm}^3$. The blue and black lines correspond to spin-up-like and spin-down-like states. The high-symmetry k-points are Γ (0, 0, 0), F (0.5, 0.5, 0), L (0.5, 0, 0), Z (0.5, 0.5, 0.5).

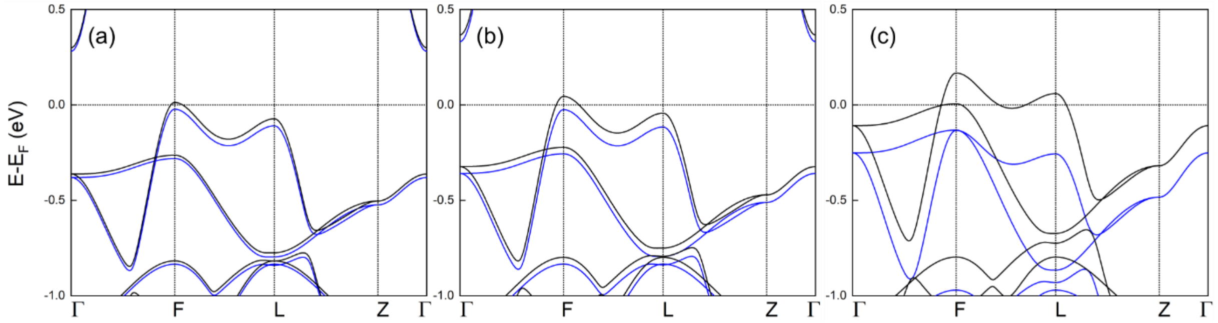

Figure S8: Electronic band structures of $CuInO_2$ unit cells at different hole densities (a) $5.0 \times 10^{20}/\mathrm{cm}^3$, (b) $1.0 \times 10^{21}/\mathrm{cm}^3$, and (c) $5.0 \times 10^{21}/\mathrm{cm}^3$. The blue and black lines correspond to spin-up-like and spin-down-like states. The high-symmetry k-points are Γ (0, 0, 0), F (0.5, 0.5, 0), L (0.5, 0, 0), Z (0.5, 0.5, 0.5).

Table S1: Cu/M^{IIIA}-O bond lengths and crystalline lattice parameters for $CuM^{IIIA}O_2$ with M^{IIIA}= Al, Ga, and In, in the delafossite structures.

	$CuAlO_2$	$CuGaO_2$	$CuInO_2$
Cu-O [Å]	1.885	1.874	1.853
M^{IIIA}-O [Å]	1.925	2.020	2.214
a=b [Å]	2.880	3.020	3.359
c [Å]	17.119	17.358	17.529
